# Neuroprotective effects of ethanol extraction from *Rubia yunnanensis* Diels on chronic cerebral hypoperfusion: modulation of the System Xc-/GSH/GPX4 axis to alleviate oxidative stress and ferroptosis

**DOI:** 10.3389/fphar.2025.1552228

**Published:** 2025-02-25

**Authors:** Jianghao Cheng, Xiaoxia Ma, Jie Tao, Xiaoli Jiang, Pu Chen, Xiaohua Duan

**Affiliations:** Yunnan Key Laboratory of Dai and Yi Medicines, Yunnan University of Chinese Medicine, Kunming, Yunnan, China

**Keywords:** *Rubia yunnanensis*, chronic cerebral hypoperfusion, System Xc-/GSH/Gpx4, serum pharmacochemistry, metabolomic, transcriptomic

## Abstract

**Introduction:**

Vascular dementia (VD) is a neurodegenerative disease caused by chronic cerebral hypoperfusion (CCH), which considerably impact patients’ quality of life. Ethanol extraction from *Rubia yunnanensis* (RY-A) has gained attention for its potential neuroprotective effects, but its effects and mechanisms of action on CCH are unknown.

**Methods:**

After 30 days of RY-A gavage treatment in a CCH rat model, its effects were evaluated using the Morris water maze test, cerebral blood flow measurements, and HE staining of the brain. These findings, combined with serum medicinal chemistry, RNA-seq, and metabolomics analyses, revealed the active compounds and mechanisms of RY-A in CCH rats. The results were further validated using assay kits and Western blot techniques.

**Results:**

RY-A treatment significantly attenuated neurological damage and improved cognitive function in CCH rats. Ultra-high-performance liquid chromatography high-resolution mass spectrometry identified 511 blood-entry compounds of RY-A. RNA-seq and metabolomic analysis showed that RY-A might help to normalize changes in gene and metabolite expression caused by CCH. RY-A induced neuroprotective effects by increasing the production of key proteins involved in ferroptosis inhibition, such as SLC7A11, SLC3A2, GSS, and GPX4, while increasing antioxidant enzyme activities and alleviating oxidative stress.

**Conclusion:**

RY-A inhibited oxidative stress and ferroptosis by activating the System Xc-/GSH/GPX4 pathway and balancing iron metabolism, thereby attenuating CCH-induced neurological damage and cognitive deficits.

## 1 Introduction

Vascular dementia (VD) is a neurodegenerative disorder associated with chronic cerebral hypoperfusion (CCH), characterized by memory impairment, cognitive decline, and behavioral changes (Pendlebury et al., 2019). With its prevalence increasing annually, VD has become a major global public health challenge, exerting a growing impact on patients and their families (Gorelick et al., 2011). Although VD is the only form of dementia that may be prevented and treated through early interventions, its complex pathomechanisms, including inflammatory responses, impaired energy metabolism, and oxidative stress, pose substantial challenges to effective treatment (Darweesh et al., 2018; Zhu et al., 2021).

Oxidative stress and lipid peroxidation play central roles in the etiology and progression of VD (Cobley et al., 2018). These processes closely resemble ferroptosis, a type of cell death caused by disruptions in intracellular iron metabolism (Hirschhorn and Stockwell, 2019). Currently, therapeutic options for VD are limited. The primary drugs, edaravone and donepezil, are often associated with side effects, such as drug resistance, liver abnormalities, and cardiac arrhythmias (Liu et al., 2023b). Therefore, the development of new treatments is crucial to improve outcomes for patients with VD.

*Rubia yunnanensis* Diels, a traditional Chinese medicinal herb, has been included in the Dian Nan Ben Cao. Its rhizomes and roots have been shown to activate the blood circulation, remove blood stasis, and soothe tendons and collaterals, as well as to have antithrombotic, anti-ischemic, and antioxidant pharmacological effects (Lan, 1959; Zhang et al., 2022). Previous studies have shown that RY-A has a protective effect against hypoxia/reoxygenation-induced damage to hippocampal HT22 cells and ameliorates oxidative stress (Cheng et al., 2024). However, the effects of RY-A on CCH and its underlying mechanisms need to be further investigated.

Metabolomics and transcriptomics, powerful tools for the study of neurodegenerative diseases, are capable of identifying and quantifying a wide range of metabolites and differentially expressed genes (DEGs), offering profound insights into the molecular underpinnings of cerebrovascular disease (Horgusluoglu et al., 2022; Arbaizar-Rovirosa et al., 2023; Askew et al., 2024). When combined with serum and plasma pharmacochemical analyses of blood-entry drug compounds, the material basis of pharmacodynamic effects can be further explored (Jin et al., 2022; Zhao et al., 2024).

The possible therapeutic impact of RY-A on CCH and its mechanism of action were examined in this study using an integrated approach involving serum pharmacochemistry, metabolomics, transcriptomics, and verification via animal experiments. Via these investigations, we aimed to establish a scientific foundation for the pharmacological effects of *Rubia yunnanensis* and offer novel perspectives on alternative preventive and treatment techniques for cerebral ischemia.

## 2 Materials and methods

### 2.1 Drug preparation

The Yunnan Key Laboratory of Dai and Yi Medicines supplied the of *Rubia yunnanensis* Yi medicine powder that was used in this investigation. Prof. Zili Yin of Yunnan University of Chinese Medicine was responsible for the drug’s authentication. The preparation process was as follows: First, 500 g of *Rubia yunnanensis* powder was taken and soaked in 95% ethanol for 24 h. Subsequently, the medicinal liquid was separated from the solid residue using a 0.22-μm polytetrafluoroethylene filter membrane. The collected medicinal liquid was concentrated by heating at 45°C using a rotary evaporator (EYELA, Shanghai, China). To ensure adequate extraction, the entire steeping and concentration process was repeated three times. The final extract obtained was *Rubia yunnanensis* ethanol extraction (RY-A), which was stored at −80°C to maintain its stability and potency.

### 2.2 Animals and treatment

The 70 male Sprague-Dawley rats, weighing 260 ± 20 g and aged 8 weeks, used in this study were supplied by SiPeiFu (Beijing Biotechnology Co., Ltd.). The rats were acclimatized and fed for 1 week, and 58 of them were operated upon by two-vessel occlusion (2VO) to establish a CCH model, while the remaining 12 served as a control group (Sham group) with bilateral carotid artery exposure only, without ligation. All animals were housed at 21°C ± 2°C, provided with adequate food and distilled water, and maintained on a 12-hour diurnal schedule. The Yunnan University of Chinese Medicine’s Ethics Committee for Animal Experimentation (Approval No. R-062022LH077) gave clearance for the use of animals in this study, and there was strict compliance with the pertinent standards of the National Institute for Animal Research.

**FIGURE 1:**
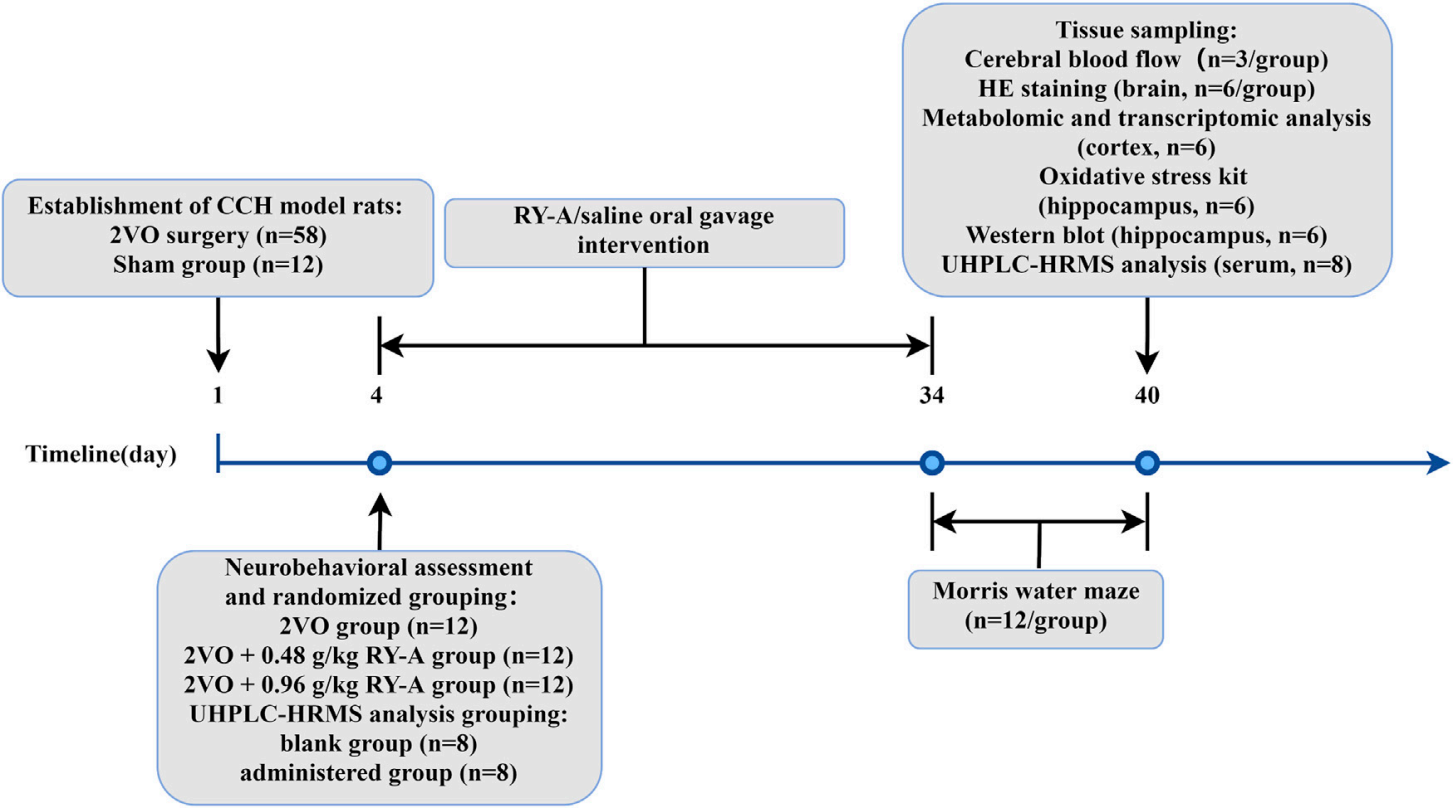
The timeline of the animal experiment of RY-A in CCH rats.

On the third day after modeling, the model rats were assessed for neurobehavioral changes using a modified 5-point scale developed by Longa et al. (1989), with model success being marked by a score of 1–3. In this study, 52 model rats were deemed successful in the model construction assessment. Thirty-six of them were randomly divided into three groups according to the random number table method: CCH model group (2VO group), RY-A low-dose group (2VO + 0.48 g/kg RY-A), and RY-A high-dose group (2VO + 0.96 g/kg RY-A). The clinical dose of RY-A was 30 g, with an extraction yield of 17.63%, and the dosages administered to the rats were calculated based on body surface area conversion (Li et al., 2024). Another 16 model rats were randomly divided into two groups of 8 rats, the 2VO group (blank group) and the RY-A high-dose group (administered group), which were used for the qualitative and quantitative analysis of RY-A blood-entry compounds. Starting from the day of scoring, rats in each group received gavage intervention with a dosing volume of 1 mL/100 g of body weight, with equal volumes of 0.9% saline given to the Sham and 2VO groups, and the treatments lasted for 30 days. The timeline of the animal experiment is shown in Figure 1.

### 2.3 Morris water maze test

The cognitive functions of learning and memory were observed using a Morris water maze after the 30-day pharmacological intervention. The apparatus consisted of a cylindrical pool containing a circular platform 30 cm high and 10 cm in diameter. The pool was filled with tap water, kept at 25°C ± 0.5°C, and colored with ink to obscure the platform from the rat’s view. Each rat was submerged in the water in the second quadrant of the pool, facing away from the platform, and the platform was placed in the fourth quadrant for testing. The main dependent variable measured was escape latency, i.e., the time a rat took to find and stay on the platform within 90 s. In case a rat failed to find the platform within the pre-specified time, the animal was guided to the platform and allowed to stay there for 10 s. Animals were trained four times a day, and the platform position was rotated clockwise to a new quadrant prior to each training session. The aforementioned training paradigm was executed for five consecutive days. A probe test was performed on the sixth day, during which the platform was removed. Rats were introduced into the pool from the second quadrant, which was situated opposite to the initial platform location, and permitted to swim freely for a duration of 120 s. Shanghai Xinruan Information Technology’s Super Maze System (Shanghai, China) was used to methodically record the behavioral data, encompassing the frequency of occurrences in the prior platform position and the duration spent in the target quadrant. These data provided us with quantitative information about the spatial memory capacity of the rats.

### 2.4 Cerebral blood flow and histological staining

After the behavioral assessment was completed, cerebral blood flow (CBF) measurements were performed on the rats. Under anesthesia, each rat’s head was fixed, and the scalp was incised and cleaned along the median line to fully expose the skull. Using a PeriCam PSI blood flow monitoring video system from Perimed, Sweden, the rat brain was illuminated by a light beam at the location where the sagittal suture meets the coronal suture. The data were processed using PIMsoft 1.5 Operational Analysis Software, which monitored the CBF in rats in a resting state for 20 s and calculated the mean value.

Brain tissue sampling was performed after CBF measurement. The animals were subjected to anesthesia, after which the thoracic cavity was perfused with 250 mL of saline, followed by 100 mL of 4% paraformaldehyde. The brains underwent postfixation for a duration of 48 h in a 4% paraformaldehyde solution, followed by embedding in paraffin. Subsequently, serial coronal sections with a thickness of 3 μm were prepared for further staining analysis. In the interim, surplus hippocampal tissue was promptly cryopreserved in liquid nitrogen and subsequently maintained at −80°C. For hematoxylin and eosin (HE) staining, paraffin-embedded slices that had been subjected to partial deparaffinization using xylene for 5 min were dehydrated via a step-by-step ethanol series (70%, 95%, and 100%, each for 5 min), and finally stained with HE. Histopathological changes in the hippocampal tissue were observed using CaseViewer 2.4 software, and images were taken at ×200 magnification for further analysis.

### 2.5 Qualitative identification of RY-A blood-entry compounds

#### 2.5.1 Extraction of RY-A samples

After transferring 600 μL of RY-A solution and 400 μL of pure methanol to a 1.5-mL EP tube, the mixture was vortexed for 10 s. Then, to 200 μL of this mixture, 200 μL of 40% aqueous methanol solution was added and vortexed to mix again for 10 s. At 4°C, after centrifugation at 16,000 × *g* for 15 min, the supernatant was collected as the extracted sample.

#### 2.5.2 Extraction of serum sample

An appropriate amount of serum sample was taken and vortexed with methanol for 60 s, then left at −20°C for 30 min. Following centrifugation at 16,000 × *g* for a duration of 20 min at 4°C, the supernatant was collected and subjected to vacuum drying. The residue was subsequently re-dissolved in 100 μL of 40% aqueous methanol, subjected to vortex mixing, and centrifuged once more. The supernatant was collected as the extracted sample (16,000 × *g*, 4°C, 15 min).

#### 2.5.3 Extraction of blank serum + RY-A sample

An appropriate amount of blank serum sample was obtained, to which RY-A solution and methanol were added. The solution was subjected to vortexing for a duration of 60 s and subsequently permitted to equilibrate at a temperature of −20°C for a period of 30 min. After centrifugation at 16,000 × *g* for 20 min at 4°C, the supernatant was obtained and subjected to vacuum drying. The dried residue was reconstituted in 100 μL of a 40% aqueous methanol solution. The extracted sample was obtained from the supernatant after further centrifugation at 16,000 × *g* for 15 min at 4°C. This procedure ensured the thorough extraction and preparation of the samples and the provision of high-quality biological specimens for subsequent analysis.

### 2.6 Ultra-high-performance liquid chromatography–high-resolution mass spectrometry analysis

#### 2.6.1 UHPLC conditions

Samples were evaluated with a Vanquish ultra-high-performance liquid chromatography–high-resolution mass spectrometry (UHPLC) system manufactured by Thermo Fisher Scientific, Germany, connected to an ACQUITY UPLC HSS T3 column with dimensions of 2.1 mm × 100 mm and a particle size of 1.8 μm. The column temperature was set at 35°C with a flow rate of 0.3 mL/min. A 0.1% aqueous solution of formic acid (A) and a 0.1% acetonitrile solution (B) were used in the two mobile phases. The gradient removal procedure below was applied by Thermo Scientific’s SII software (version 1.4): the concentration of phase B commenced at 5%, escalating to 25% after 3 min, 45% after 8.5 min, 95% after 14 min, and reaching 98% at 17 min. Between 17 and 17.2 min, the B-phase fraction was decreased to 5% until the 20 min mark.

#### 2.6.2 HRMS conditions

Q-exactive HFX mass spectrometry analysis was conducted to provide primary and secondary spectra for the samples. The mass spectrometer was connected to the UHPLC system, utilizing ESI (+/−) for the acquisition of the mass spectrum. The spray voltage was established at 3800 V (ESI+) and 3500 V (ESI−), the sheath gas pressure was maintained at 45 arb, the auxiliary gas pressure was set to 20 arb, the ion transfer tube temperature was regulated to 320°C, and the nebulization temperature was adjusted to 350°C. Detection occurred in full-MS/data-dependent secondary scanning (full-MS/dd-MS2) modes, with first-order and second-order resolutions of 60,000 and 15,000, respectively. The 10 highest-intensity MS1 ions were chosen for MS/MS spectrum capture, with collision energies set at normalized values of 20, 40, and 60 units. The principal mass-to-charge ratio scanning range was 90 m/z to 1,300 m/z.

#### 2.6.3 Sample detection and analysis

Then, 6 μL of each blank group sample, administered group sample, blank group + RY-A sample, and RY-A solution were injected separately for liquid chromatography–mass spectrometry analysis. One injection of each batch of blank group samples and administered group samples was added, with repeated injections (3 for blank group + RY-A samples, 5 for RY-A samples). The proteoWizard software package was used to import the raw files (.raw) and convert them into the “.mzXML” format. Peak alignment, retention time adjustment, and peak extraction were performed with the XCMS program. The compounds were identified by querying the high-resolution mass spectrometry database of Shanghai Applied Protein Technology, with a primary mass error of less than 25 ppm and a secondary fragmentation spectrum match score exceeding 0.7. A higher score indicates greater spectral similarity, and a score of 0.7 or above is typically regarded as a dependable identification outcome.

### 2.7 Transcriptomic analysis

Total RNA was extracted from the left cortical region of rats using the Trizol method (Joy et al., 2018). Extracted RNA samples were solubilized in diethylpyrocarbonate-treated water, then analyzed qualitatively and quantitatively using a Qubit fluorescence quantimeter from Thermo Fisher Scientific (MA, USA) and a Qsep400 high-throughput biofragment analyzer from BiOptic Inc. (Taiwan, China). The mRNA with polyA tails were enhanced using Oligo (dT) beads with magnets, then fragmented at the specified temperature. These fragments served as templates for reverse transcription utilizing random hexamer primers to generate the first-strand cDNA, followed by the synthesis of the second-strand cDNA. dUTP was employed instead of dTTP in this process to achieve the doping of the second strand of the cDNA and the repair of the double-stranded cDNA ends. Next, sequencing adapter ligation was performed, and DNA magnetic bead purification and fragment selection were used to obtain 250-bp to 350-bp insert fragments, then PCR amplification was performed. The constructed libraries were quality checked on the Qubit instrument and Qsep400 instrument and were accurately quantified to assure the effective Q-PCR concentration, which is usually considered to be greater than 2 nM. Metware Biotechnology Co. Ltd. (Wuhan, China) used the Illumina platform to sequence the qualifying libraries. HISAT2 version 2.2.1 software (https://daehwankimlab.github.io/hisat2/) was used to match clean reads to the reference genome sequence. Gene comparisons were conducted utilizing the featureCounts tool, while gene expression levels were assessed by the fragments per kilobase of transcript per million fragments mapped (FPKM) methodology. Then, the transcriptomes of the RY-A group and 2VO group were compared with DESeq2, with three biological replicates of each group. Genes were categorized as DEGs if their corrected *p*-value was less than 0.05 and their |log2 fold change (FC)| was more than 1. Ultimately, the clustering procedure was performed to elucidate the expression patterns of the DEGs across various experimental settings utilizing the R package. The enrichment of DEGs was studied using a hypergeometric test. For KEGG pathway (http://www.genome.jp/kegg/) and GO annotation (http://www.ebi.ac.uk/QuickGO/), the hypergeometric distribution test and GO term analysis were performed, respectively.

### 2.8 Metabolomic analysis

To thoroughly examine RY-A’s metabolic effects on the CCH rat model, metabolomic analysis was performed on the right cortical region of rats from the RY-A and 2VO groups. Initially, six samples from each group were homogenized for 20 s in a ball mill at a frequency of 30 Hz. Centrifugation was used to sediment the samples for 30 s at 4°C and 3,000 × *g*. Four hundred microliters of a methanol-water internal standard extract were added to the samples, shaken at 2,500 revolutions per minute for 5 min, and allowed to stand on ice for 15 min. Then, at the same temperature as used previously, the samples were centrifuged for 10 min at 12,000 × *g*. After 30 min, 300 µL of the supernatant was removed and kept at −20°C. Following 3 minutes of centrifugation at 12,000 × *g*, 200 µL of the supernatant was taken for analysis by mass spectrometry.

The UPLC parameters were delineated as follows: The column employed was a Waters ACQUITY UPLC HSS T3 C18 (1.8 µm, 2.1 mm × 100 mm); mobile phase A consisted of a water solution containing 0.1% formic acid, whereas mobile phase B comprised acetonitrile with 0.1% formic acid. The flow rate was 0.4 mL/min, and the injection volume was 2 μL. The column temperature was sustained at 40°C. Gradient program: Phase B was begun at 5%, increased to 20% at 2.0 min, reached a maximum of 99% at 6.0 min and continued until the 7.5 min mark, then lowered to 5% at 7.6 min and continued to 10.0 min. This analysis was conducted by Metware Biotechnology Co., Ltd.

In the pre-processing stage, the data underwent unit variance scaling. Orthogonal partial least squares discriminant analysis was conducted using OPLSR, an analytical function within the MetaboAnalystR package of R. Metabolites were designated differentially expressed metabolites (DEMs) when their VIP values were above 1 and their *p*-values were below 0.05, as determined by Student’s t-test within the orthogonal partial least squares discriminant analysis (OPLS-DA) model. The expression patterns of DEMs were analyzed by clustering, with heatmaps drawn in the R package. Metabolic pathways were annotated for DEMs by utilizing the KEGG database. These analytical steps provided us with a comprehensive view of the metabolic effects of RY-A on the CCH rat model, revealing the underlying metabolic regulatory mechanisms.

### 2.9 Joint analysis of the transcriptome and metabolome

Based on the transcriptomic and metabolomic data, the KEGG signaling pathways in which the DEGs and DEMs were co-enriched were further analyzed. The Pearson correlation coefficients for comparisons between gene expression variations and metabolite alterations were computed using the cor function in the R program. Subsequently, relationships with an absolute Pearson correlation coefficient over 0.8 and a *p*-value below 0.05 were identified, and a nine-quadrant plot was constructed.

### 2.10 Oxidative stress kit assay

The subsequent steps were undertaken to assess the degree of oxidative stress in hippocampal tissue.

#### 2.10.1 Reactive oxygen species level assay

Fifty milligrams of hippocampal tissue samples were mixed thoroughly with 1 mL of homogenization buffer A. Following sediment removal using centrifugation, the supernatant was obtained. Two hundred microliters of the supernatant and 2 µL of the DHE probe were put into a 96-well plate, thoroughly mixed, and incubated at 37°C for 30 min under light protection. The fluorescence intensity was measured by setting the FLIR’s excitation and emission wavelengths to 488–535 nm and 610 nm, respectively. After taking out 50 µL of the supernatant, 100 µL was used for protein quantification after 30-fold dilution with PBS. Reactive oxygen species (ROS) levels were expressed as the fluorescence intensity divided by milligrams of protein.

#### 2.10.2 Detection of malondialdehyde, superoxide dismutase and glutathione peroxidase levels

We sampled 50 mg of hippocampal tissue and mixed it thoroughly with 1 mL of homogenization buffer A. The samples were centrifuged and the supernatant collected. The instructions that came with the malondialdehyde (MDA), superoxide dismutase (SOD), and glutathione peroxidase (GSH-Px) kits were followed. A thiobarbituric acid assay was used to measure MDA levels, the WST-8-based colorimetric reaction was applied to measure SOD activity, and the DTNB method was used to measure GSH-Px protein levels.

### 2.11 Western blot analysis

Western blot was employed to evaluate the proteins in rat hippocampal tissues linked to iron metabolism and the System Xc-/GSH/GPX4 pathway. Six rats in each group had their hippocampal tissues quickly frozen in liquid nitrogen and kept at −80°C after all animals were sacrificed. The hippocampal tissues were lysed using RIPA lysing solution (KGP702-100; KeyGEN Bio TECH, NanJing, China) containing PMSF (97064-898; Amresco, VWR International, OH, USA), and were thoroughly mixed by an electric homogenizer. The supernatant was obtained using centrifugation at 12,000 × *g* for 5 min at 4°C, and the protein content was assessed using the BCA Protein Quantification Kit (KGP902; KeyGEN Bio TECH).

Gel electrophoresis was conducted to transfer the proteins onto a PVDF membrane, which was subsequently sealed with a TBST solution containing 5% skim milk powder. After that, the following antibodies were used: transferrin receptor antibody (1:5000; ab269513), anti-SLC7A11 antibody (1:1000; ab307601), anti-β-actin antibody (1:1000; ab8227), goat anti-rabbit IgG H&L (HRP) (1:10000; ab6721), and rabbit anti-mouse IgG H&L (HRP) (1:10000; ab6728), all from Abcam; anti-ferroportin/SLC40A1 antibody (1:500; 26601-1-AP) from Proteintech; and anti-CD98/SLC3A2 antibody (1:200; sc-136139), anti-GPX-4 antibody (1:100; sc-166120), and anti-GSS antibody (1:100; sc-365863) from Santa Cruz Biotechnology. Subsequent to the initial incubation, a further incubation of 1 h at an ambient temperature was conducted utilizing the appropriate secondary antibodies. The software Image Pro Plus 6.0 was used to capture the optical density values of the protein bands. Then, the proteins’ relative expression levels were ascertained by dividing the target protein’s grayscale values by those of the internal reference protein.

### 2.12 Molecular docking

Molecular docking experiments were used in this study to explore at how the possible active chemicals in RY-A interacted with the main targets. Initially, the identifiers of the primary targets were retrieved from the UniProt database, and their three-dimensional structures were acquired from the Protein Data Bank (PDB, https://www.rcsb.org/). Concurrently, the three-dimensional structures of the prospective active chemicals in RY-A were acquired from the PubChem database and stored in SDF format. These structures underwent pre-processing with PyMOL software (https://pymol.org/2), which involved the elimination of water molecules and non-essential small-molecule ligands from the structures. Hydrogen atoms were included, and the charges were allocated to these structures via AutoDock Tools (https://autodocksuite.scripps.edu/adt/). The processed structures were then saved in PDBQT format to enable molecular docking simulations.

Molecular docking simulations were conducted utilizing Vina 2.0 software, designating the key target proteins as receptors and the prospective active chemicals in RY-A as ligands. Evaluating the binding energy allowed for the prediction of the interaction intensity between these active molecules and their targets. The analysis of binding energies showed that compounds with binding energies below −4.25 kcal/mol had possible binding activity, those with energies below −5.0 kcal/mol had stronger binding activity, and compounds with energies below −7.0 kcal/mol had considerable binding activity (Hsin et al., 2013). Upon completion of docking, the findings were visualized and analyzed in three dimensions with PyMOL software, while two-dimensional plots were produced using Discovery Studio 2020 Client software.

### 2.13 Statistical methods

Statistical analysis of the data in this study was performed using GraphPad Prism 9.0 software, and the results are expressed as the mean ± standard error of the mean. To examine the differences among the various experimental groups, one-way analysis of variance was employed. In this analysis, differences were deemed statistically significant when the *p*-value was below 0.05.

## 3 Results

### 3.1 Improvements in spatial learning and memory ability in RY-A-treated CCH rats

**FIGURE 2:**
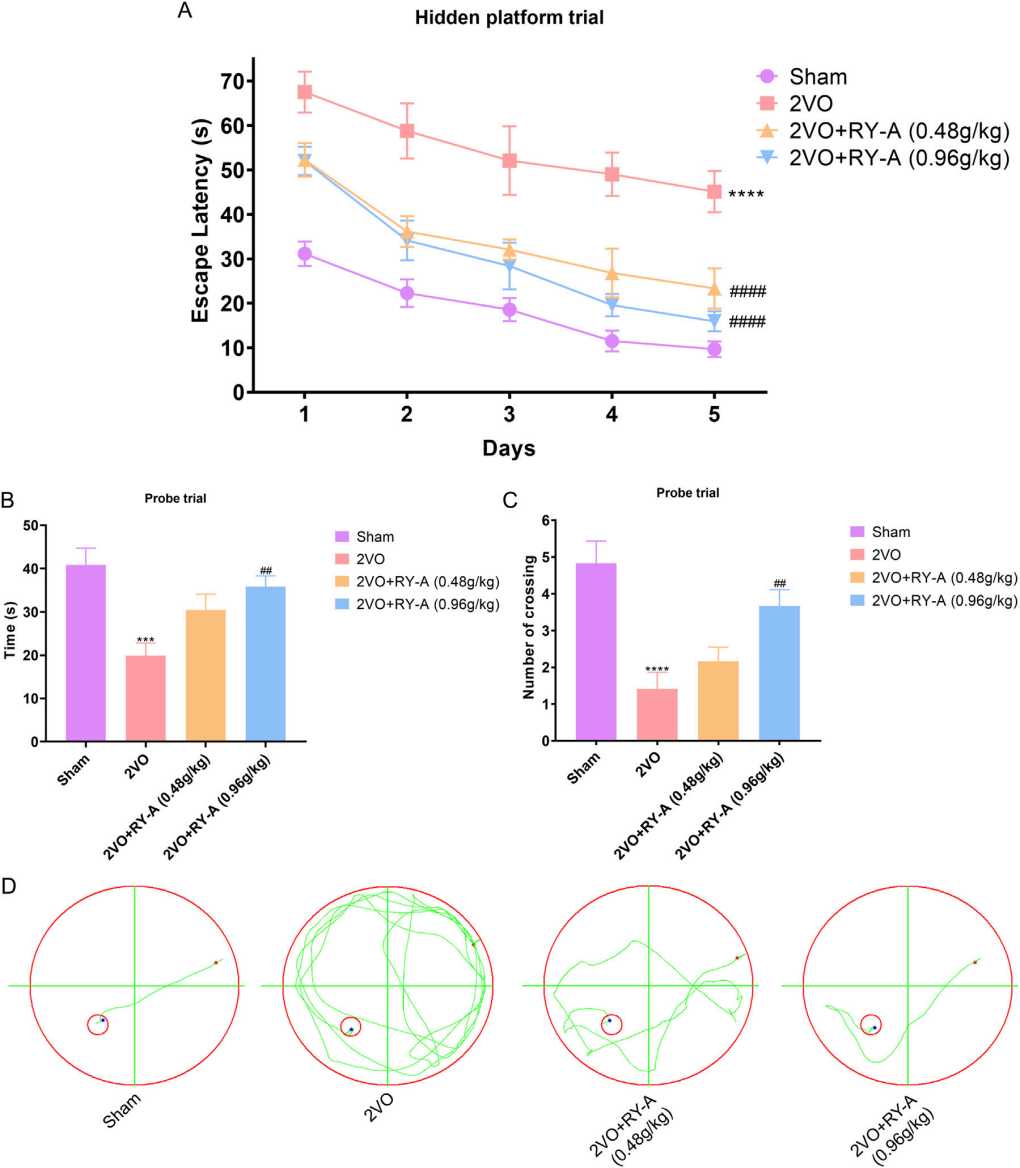
RY-A enhances the learning and memory capacities of CCH rats in the Morris water maze test. **(A)** Average escape latency for every rat group in the concealed platform experiment. **(B)** Each group’s swimming time during the probe trial in the quadrant containing the original platform. **(C)** The number of times throughout the probe experiment that each group crossed the initial platform. **(D)** Swim trajectory charts that are representative of each rat group. Data are expressed as mean ± SEM (n = 12). Compared to the Sham group, ****p < 0.0001; Compared to the 2VO group, ^*##*^ *p* < 0.01, ^*####*^ *p* < 0.0001. 2VO, 2-vessel occlusion; RY-A, *Rubia yunnanensis* alcohol extract.

CCH has been shown to cause impairments to of spatial memory ability (Yuan et al., 2019). This study assessed the possibility of RY-A enhancing the learning and memory capabilities of CCH rats using the Morris water maze test, and the experimental findings are illustrated in Figure 2. In the concealed platform assessment, the mean escape delay was documented for each cohort of rats during a 5-day duration. The findings indicated (Figures 2A, D) that the average escape delay across all groups diminished with training. The 2VO group exhibited a significantly prolonged mean escape latency relative to the Sham group, signifying their compromised learning and memory capabilities (P < 0.0001). Following RY-A therapy, rats in the low and high RY-A dosage groups exhibited markedly reduced escape latency in comparison to the 2VO group (0.48 g/kg RY-A: P < 0.0001; 0.96 g/kg RY-A: P < 0.0001), indicating RY-A substantially enhanced the rats’ learning and memory capabilities.

During the probe test, all rats exhibited a tendency to swim in the quadrant where the initial platform was situated. Figures 2B, C illustrate that, after 33 days of chronic ischemia treatment, rats in the 2VO group exhibited a significant reduction in the time spent swimming in the original platform and the frequency of crossing the original platform compared to the Sham group (P < 0.001). These results highlight the detrimental effects of CCH on spatial memory capabilities. Conversely, RY-A therapy greatly reversed these changes. Rats in the treatment group spent more time swimming in the original platform quadrant (P < 0.0001) and crossed the original platform more often (0.96 g/kg RY-A: P < 0.01). These findings indicate that RY-A markedly enhanced spatial learning and memory impairments in CCH rats.

### 3.2 Effect of RY-A on increasing CBF and improving of neuronal morphology in hippocampal and cortical regions in CCH rats

**FIGURE 3:**
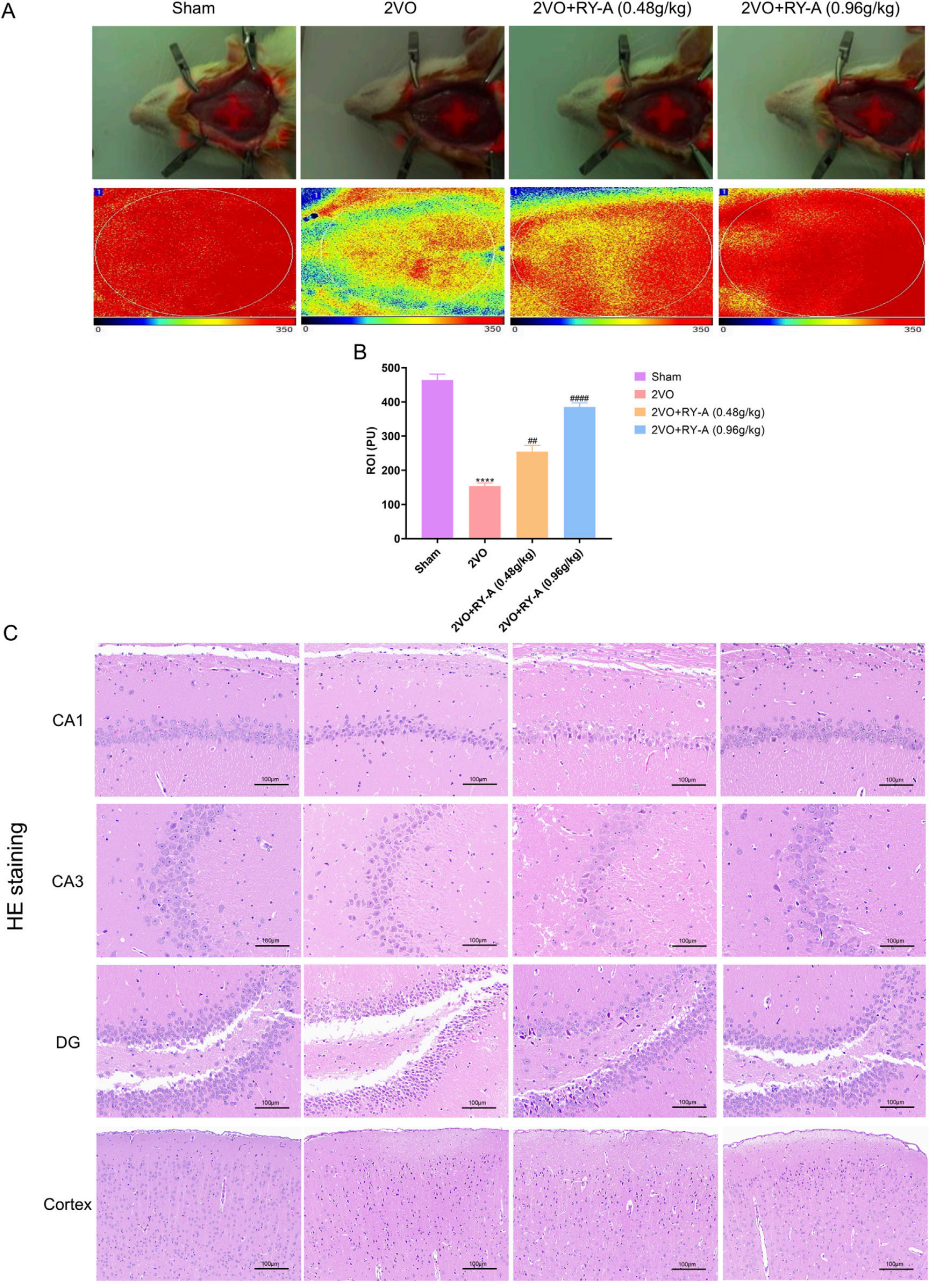
Effects of RY-A therapy on cortical, DG, CA1, CA3, and CBF neuronal morphology in CCH rats. **(A, B)** Effects of RY-A therapy on CBF in VaD rats (n = 3). **(C)** HE staining’s effects on the morphology of neurons in the DG, CA1, CA3, and cortical regions of the VaD rats (200 x, n = 6). Data are expressed as mean ± SEM. Compared to the Sham group, ****p < 0.0001; Compared to the 2VO group, ^*##*^ *p* < 0.01, ^*####*^ *p* < 0.0001. 2VO, 2-vessel occlusion; RY-A, *Rubia yunnanensis* alcohol extract.

The CBF of rats was measured in each group using a laser scattering technique to assess the effect of RY-A in CCH rats. As shown in Figures 3A, B, CBF was significantly lower in the 2VO group than the Sham group (p < 0.0001), suggesting that CCH resulted in reduced CBF. After RY-A treatment, CBF was significantly enhanced, with the most significant effect seen in the RY-A high-dose group (0.48 g/kg RY-A: p < 0.01; 0.96 g/kg RY-A: p < 0.0001), which suggests that RY-A is effective in ameliorating the reduction in CBF caused by CCH.

Histological examination is a key method for assessing nerve cell damage and drug efficacy (Brun and Englund, 1986). Neuronal abnormalities in CA1, CA3, DG, and cortical areas of the hippocampal region are closely associated with spatial learning disabilities. Using HE staining, the loss of neurons in these regions was observed in the brain tissue of rats after modeling and the degree of recovery after RY-A treatment was recorded. As shown in Figure 3C, the 2VO group’s cerebral cortex and hippocampus had considerably lower numbers of neurons than those of the Sham group, along with larger cell gaps and a disorganized cell layout. Treatment with RY-A significantly improved these pathological changes and attenuated the CCH-induced neural tissue damage. This is consistent with the trends observed in the behavioral experiments. Therefore, the RY-A high-dose group and 2VO group were selected for subsequent transcriptomic and metabolomic studies. In addition, the above data suggest that RY-A has potential therapeutic effects on CCH-induced neurological damage and cognitive deficits by promoting CBF and improving neuronal morphology.

### 3.3 Identification of blood-entry compounds in RY-A using UHPLC-HRMS technique

**FIGURE 4:**
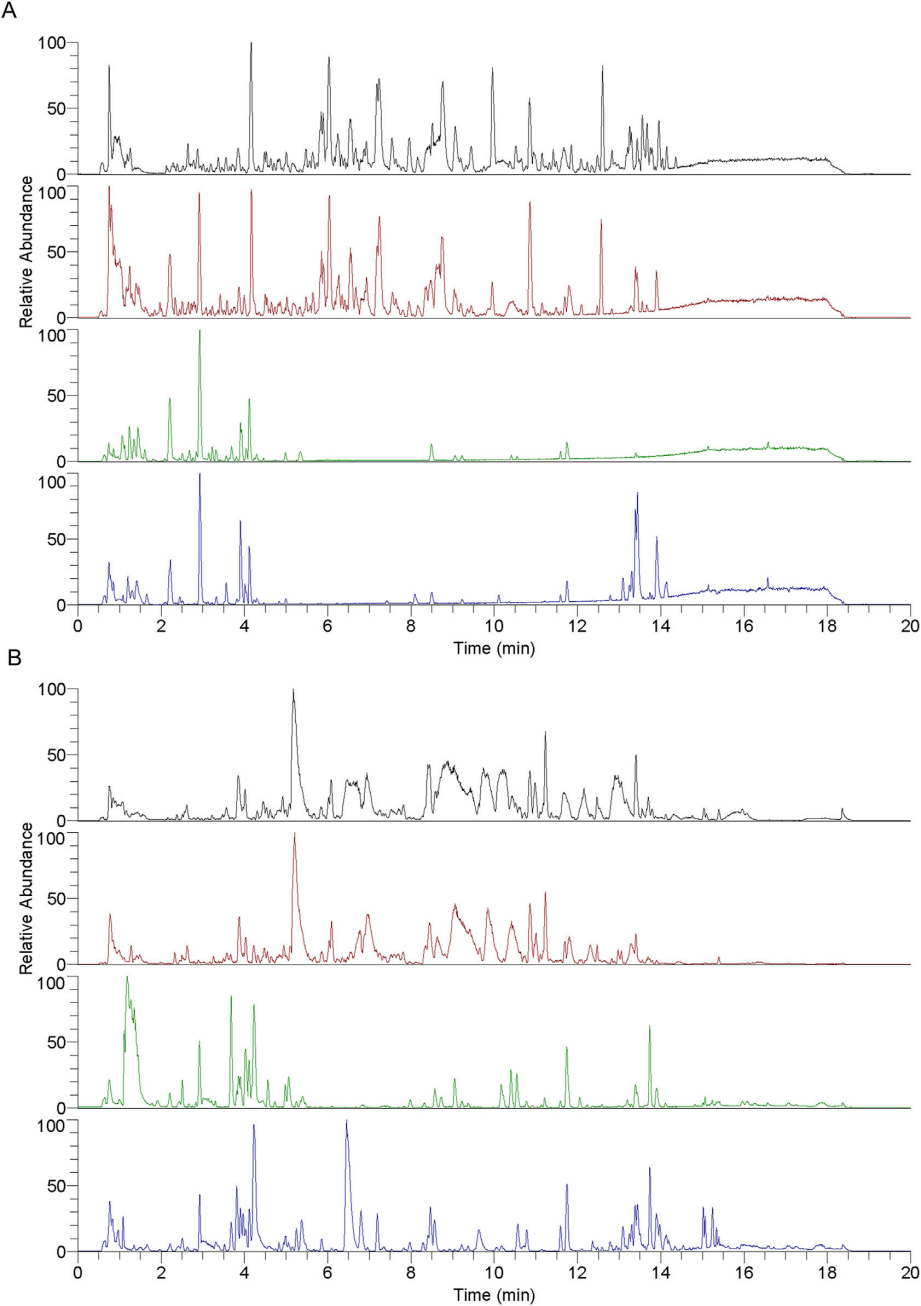
Each group of samples’ base peak chromatograms (BPC) as determined by UHPLC-HRMS analysis. **(A)** Positive ion scan and **(B)** negative ion scan. Top to bottom, the chromatograms show the following: RY-A sample, blank group (2VO group) + RY-A sample, blank group, and RY-A high-dose group (administered group).

A detailed analysis of the blood-entry compounds of RY-A was performed by UHPLC-HRMS. Figure 4 illustrates the base peak chromatogram of each group of samples, while Supplementary Table S1 lists our preliminary identification of 511 blood-entry compounds of RY-A, including 276 compounds identified in the positive ion mode and 240 compounds identified in the negative ion mode. It has been shown that triterpenoids and quinones are key active *Rubia yunnanensis* compounds against hyperlipidemia, which is one of the key pathological factors of CCH (Gao et al., 2014). Five quinones and triterpenoids were deduced from the literature review, and the known standards rhein (Liu et al., 2023a), emodin 8-glucoside (Wang et al., 2007), alisol A (Li et al., 2023), dihydrotanshinone (Wu et al., 2023), and corosolic acid (Zhang et al., 2024) are potential active compounds in RY-A with neuroprotective effects.

**FIGURE 5:**
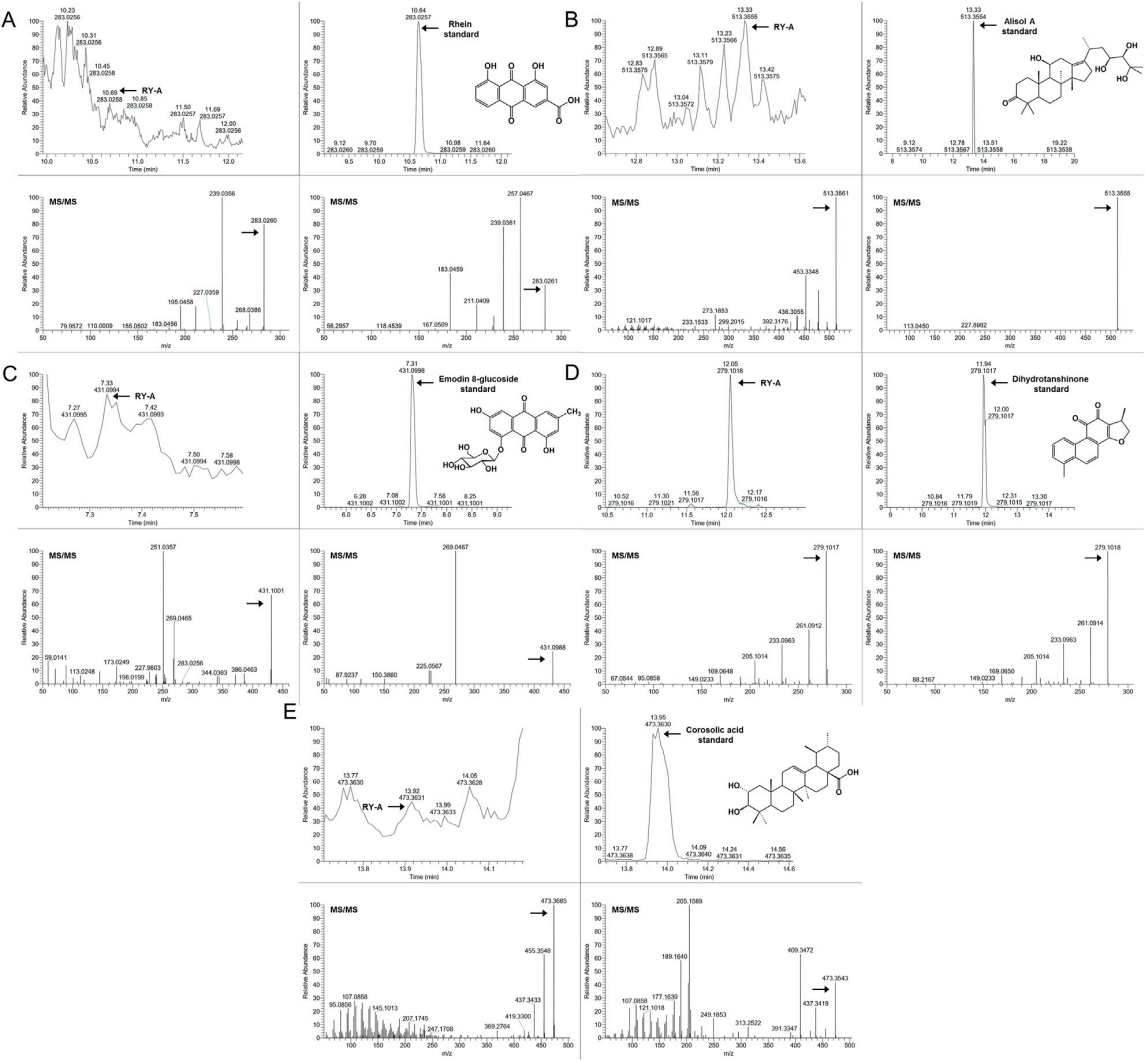
The administered group’s specific extracted ion chromatogram (XIC) and secondary mass spectra in comparison to established standards. **(A)** Rhein, **(B)** Alisol A, **(C)** Emodin 8-glucoside, **(D)** Dihydrotanshinone, **(E)** Corosolic acid.

In order to verify the entry of these compounds into the blood, serum samples from the RY-A-administered group were further analyzed and compared using the retention time and MS/MS fragment information of the known standards. This step confirmed the accuracy of our UHPLC-HRMS results. Figure 5 demonstrates the specific extracted ion chromatograms and secondary mass spectra of these standards, which provide the material basis for the pharmacological effects of RY-A.

### 3.4 DEGs screening results

**FIGURE 6:**
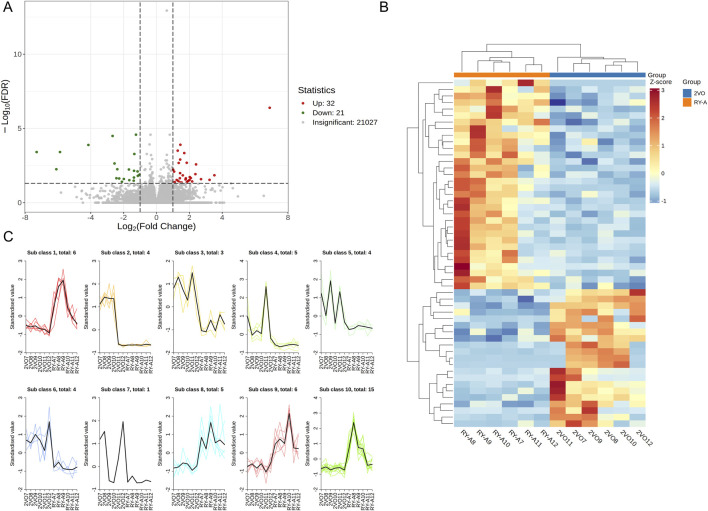
DEGs of RY-A and 2VO groups. **(A)** Volcano plot of DEGs. **(B)** Hierarchical clustering of DEGs. **(C)** Kmeans clustering of DEGs.

In this study, a total of 50.2 Gb of raw data was obtained by transcriptome sequencing. The amount of sequencing data per sample ranged from 1.61 Gb to 2.33 Gb, of which 97.73%–98.16% met the Q20 standard, indicating high sequencing accuracy. In addition, 93.2%–94.37% of the data quality met the Q30 standard with an average GC content of 50.79%. By comparing the RY-A group with the 2VO group, DEGs were screened with a corrected *p*-value ≤0.05 and |log2 FC| ≥ 1 (Figure 6A). Using FPKM values, hierarchical clustering and Kmeans clustering analyses were performed on the DEGs to analyze differences in the gene expression patterns (Figures 6B, C). A total of 32 genes upregulated and 21 genes downregulated in expression were identified under RY-A treatment (Supplementary Table S2). Among them, ENSRNOG00000031607 gene expression was most significantly downregulated, whereas ENSRNOG00000065367 gene expression was most significantly upregulated, and their log2 FC values were −7.29 and 6.89, respectively.

### 3.5 DEGs functional and pathway enrichment analysis results

**FIGURE 7:**
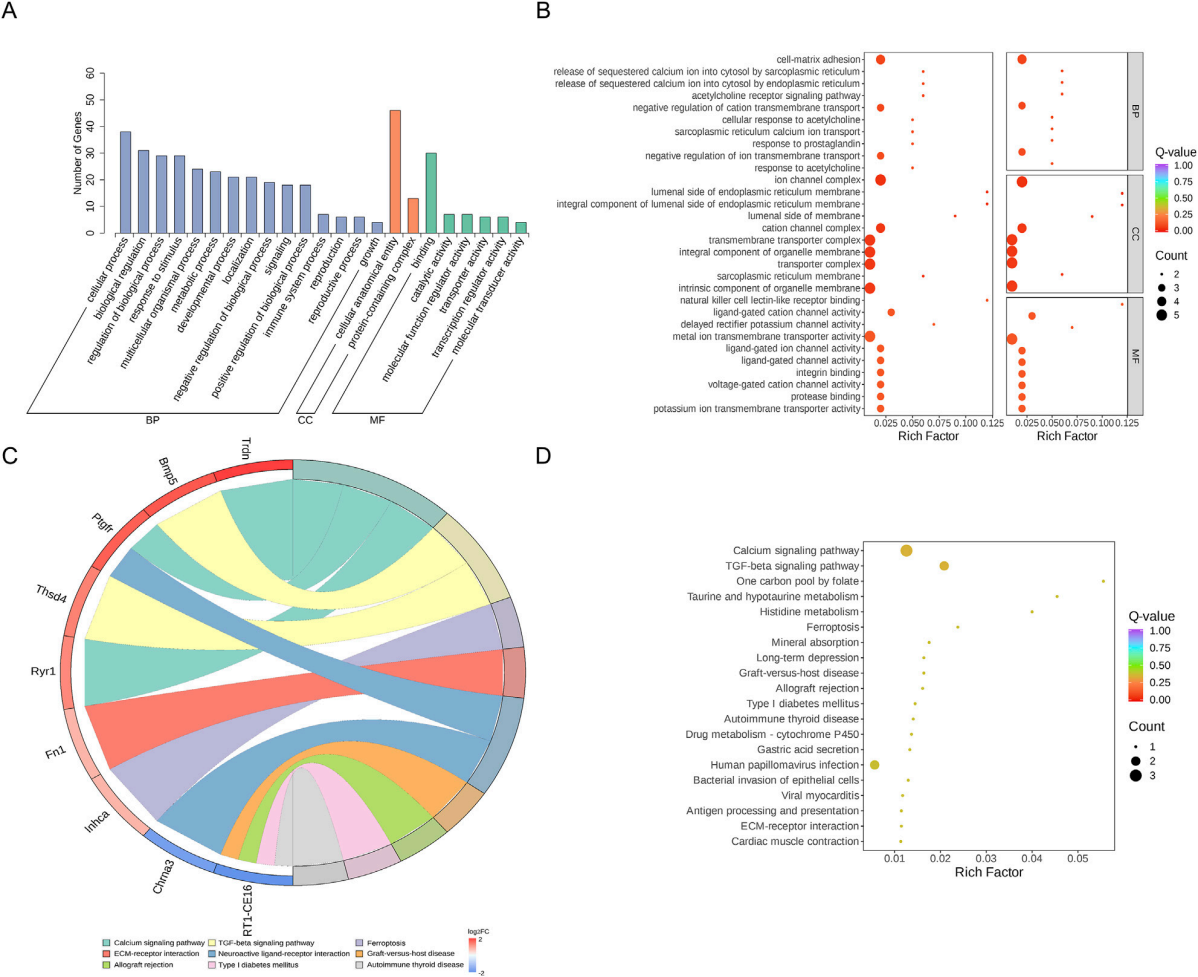
GO and KEGG enrichment analysis of DEGs. **(A)** DEGs’ GO categorization histogram. **(B)** DEG GO enrichment scatter plot. **(C)** KEGG enrichment chord plot of DEGs. **(D)** DEGs in a KEGG-enriched scatter plot.

The DEGs were also analyzed using GO and KEGG enrichment analyses. GO enrichment analysis classified the DEGs into three functional categories: biological process (BP), cellular component (CC), and molecular function (MF). Supplementary Table S3 lists the 1,027 BPs, 128 cellular components, and 183 molecular functions that were stronger in the 2VO group than the RY-A group. Most of the expressed genes were allocated to three groups: biological regulation (GO:0065007), control of BP (GO:0050789), and cellular process (GO:0009987) (Figure 7A; Supplementary Table S4). The 10 best categories in BP, CC, and MF were chosen for the bubble plot (Figure 7B), and genes were arranged in order of the Q-value (corrected *p*-value). The findings indicated that the majority of the genes were enriched in activities that included the negative regulation of cation transmembrane transport (GO:1904063), ion channel complex (GO:0034702), and metal ion transmembrane transporter activity (GO:0046873).

KEGG enrichment analysis revealed the three pathways most significantly altered between the RY-A group and the 2VO group: calcium signaling pathway (rno04020, *p*-value = 0.0079), TGF-beta signaling pathway (rno04350, *p*-value = 0.012), and one carbon pool by folate pathway (rno00670, *p*-value = 0.031) (Figure 7D). In addition, significant changes were observed in the pathways taurine and hypotaurine metabolism (rno00430, *p*-value = 0.038), histidine metabolism (rno00340, *p*-value = 0.043), and ferroptosis (rno04216, *p*-value = 0.044) (Supplementary Table S5). The chord plot demonstrates the nine pathways with the lowest p-values (Figure 7C), and the upregulated genes with the largest multiplicity of differences on these pathways were Trdn, Bmp5, Ptgfr, Thsd4, Ryr1, Fn1, and Inhca; while the downregulated genes were Chrna3 and RT1-CE16. These enrichment analyses suggested that these pathways and genes may be closely related to the pathogenesis of CCH and the therapeutic efficacy of RY-A.

### 3.6 Results of metabolomic analysis

**FIGURE 8:**
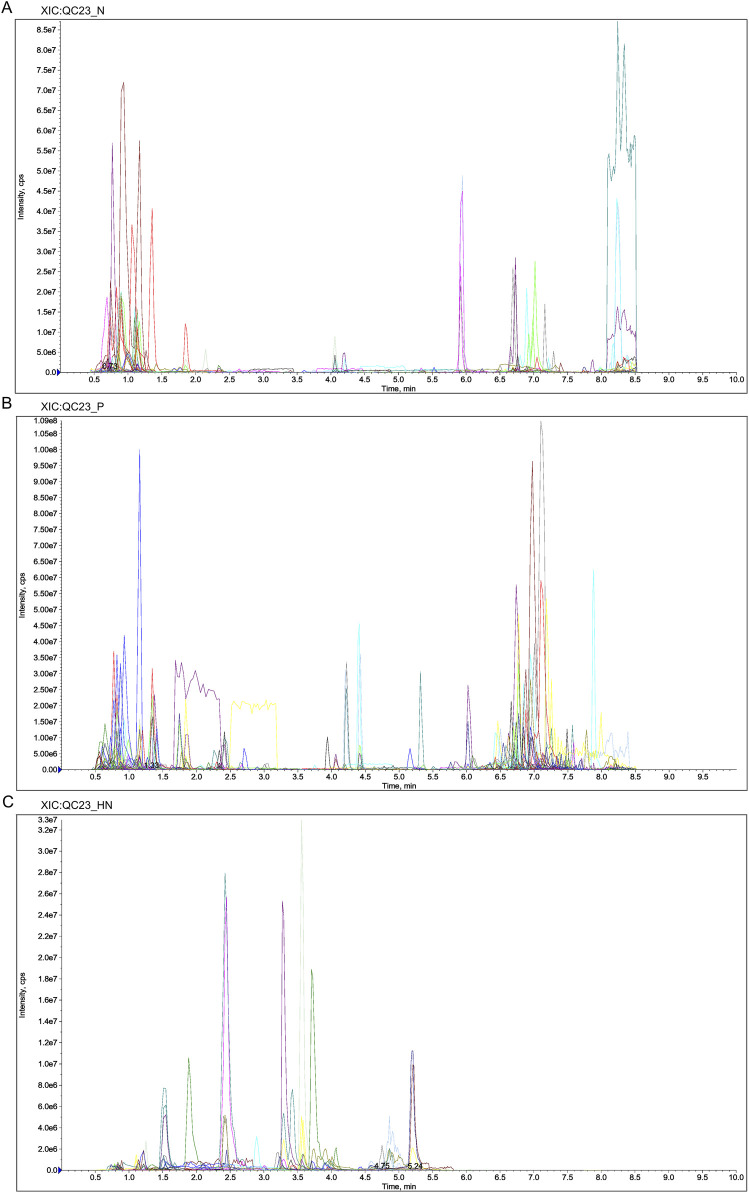
Extracted Ion Chromatogram (XIC) of MRM. **(A)** XIC of N. **(B)** XIC of P. **(C)** XIC of HN. Abbreviations: N, T3 negative ion mode; P, T3 positive ion mode; HN, HILIC negative ion mode.

**FIGURE 9:**
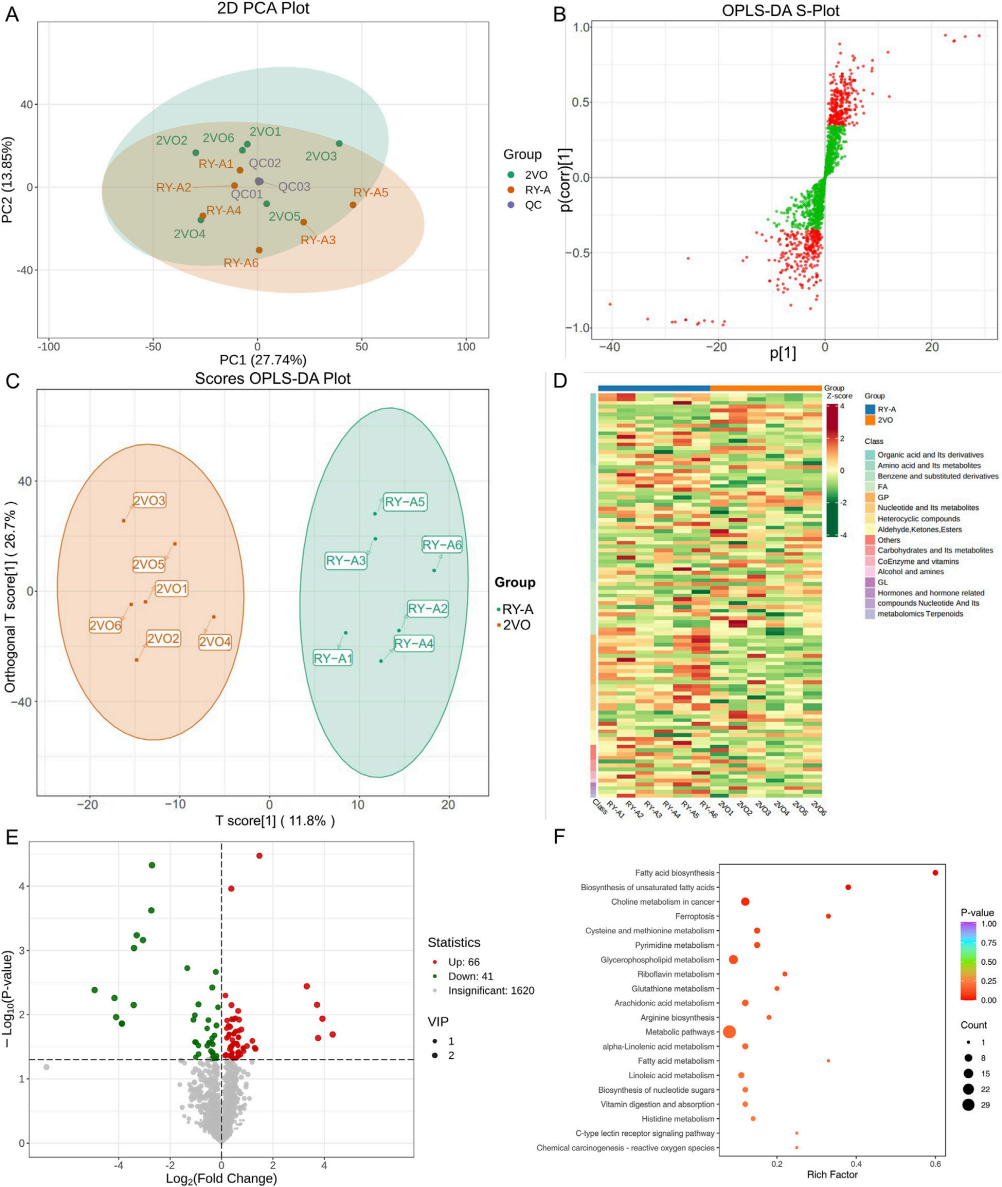
Analysis of RY-A and 2VO groups’ DEMs. **(A)** Analysis of PCA. **(B)** The OPLS-DA S-Plot. **(C)** Plot of OPLS-DA Scores. **(D)** Clustering Heatmap of DEMs. **(E)** Volcano Plot of DEMs. **(F)** KEGG enrichment plot of DEMs.

Liquid chromatography-quadrupole time-of-flight mass spectrometry was utilized in this investigation to identify metabolites in the samples. The metabolites were measured in pre-processing via mass spectrometry peak extraction, calibration and annotated for metabolite identification, followed by quantitative analysis utilizing triple quadrupole mass spectrometry in multiple reaction monitoring mode. A total of 1,727 metabolites were identified, comprising 830 in the positive ion mode and 897 in the negative ion mode (616 in the T3 negative ion mode and 281 in the HILIC negative ion mode) (Figures 8A–C). The findings of principal component analysis showed that the two sample groups’ metabolites differed significantly (Figure 9A). The score plots and S-plots of the OPLS-DA models further validated the significant variations in metabolomics between the groups and the absence of outlier data points (Figures 9B, C).

Clustering analysis of 107 DEMs based on the Z-score values identified 41 metabolites with downregulated expression between the RY-A and 2VO groups, including L-fucose (VIP = 2.85, p = 0.000047), trolox (VIP = 2.84, p = 0.00092), and 3-(imidazol-4-yl) propionic acid (VIP = 2.81, p = 0.0071), and the upregulation of 66 metabolites, such as 12-oxoETE (VIP = 2.73, p = 0.012), GSH (VIP = 2.65, p = 0.023), and adrenic acid (AA, VIP = 1.96, p = 0.012) (Figures 9D, E). These DEMs were mostly engaged in ferroptosis (ko04216), glutathione metabolism (ko00480), fatty acid biosynthesis (ko00061), and unsaturated fatty acid biosynthesis (ko01040), among other BPs, according to the results of the KEGG enrichment studies (Figure 9F; Supplementary Table S6).

### 3.7 Results of transcriptome and metabolome integration analysis

**FIGURE 10:**
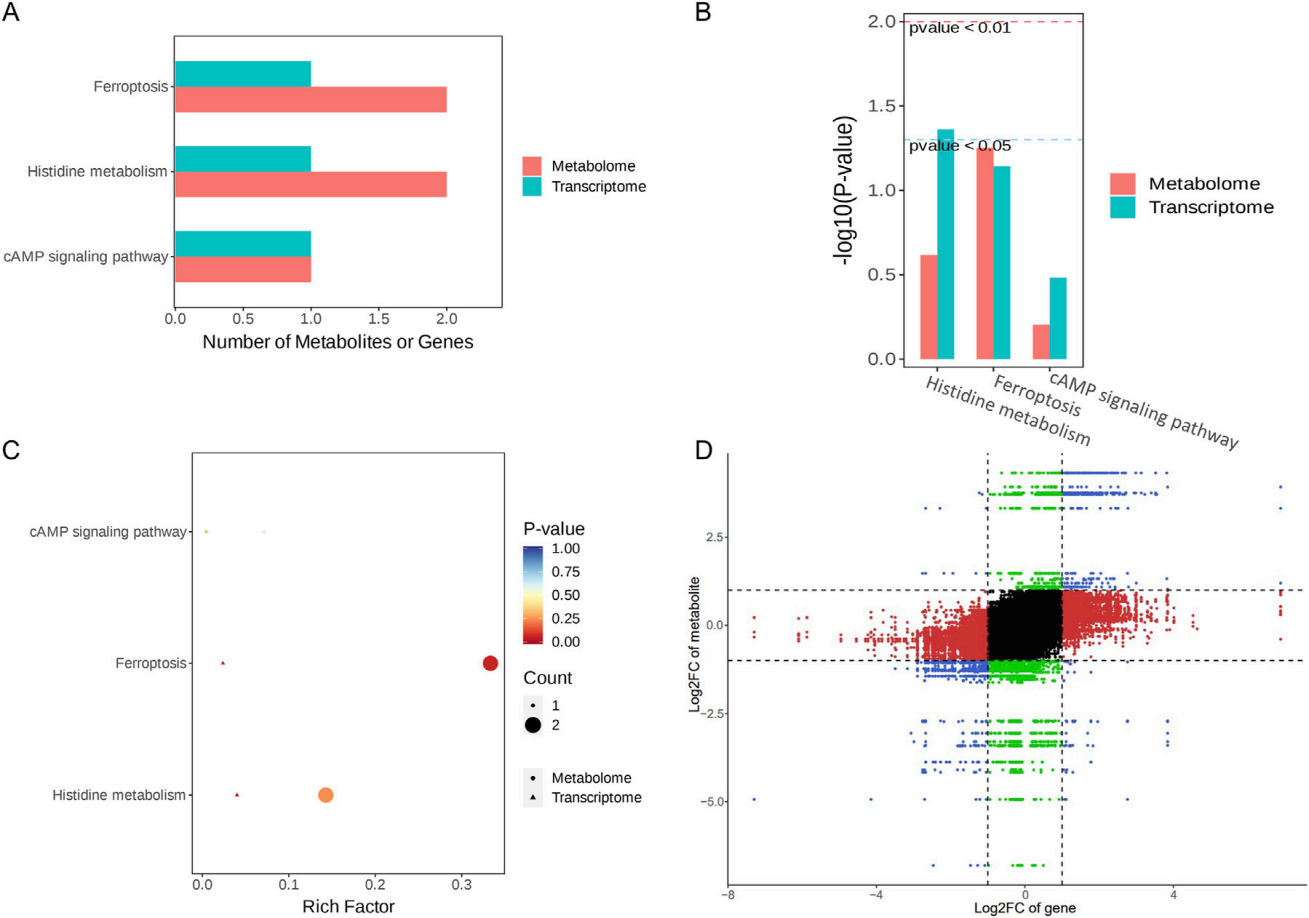
Integration analysis of the transcriptome and metabolome. **(A)** KEGG Enrichment Bar Plot. **(B)** KEGG Enrichment Column Plot. **(C)** KEGG Enrichment Bubble Plot. **(D)** Nine- quadrant Plot.

In a comparison of the RY-A group and 2VO group, 53 DEGs and 107 DEMs were identified. Ferroptosis (ko04216), histidine metabolism (ko00340), and the cAMP signaling pathway (ko04024) were found to be co-enriched in the integration study of the two omics datasets, according to KEGG pathway analysis (Figures 10A–C). This implies that these routes could be the main mechanisms by which RY-A produces its pharmacological effects. Genes linked to the integration analysis, including RGD1310507 and Hcar1, showed considerably higher expression in the RY-A group, although Ftcd expression was significantly lower. Similarly, metabolites such as GSH, AA, 3-hydroxybutanoic acid, and [1-(5-phosphoribosyl) imidazol-4-yl] acetic acid were significantly increased in the RY-A group, whereas 3-(imidazol-4-yl) propionic acid 3-(imidazol-4-yl) propionic acid was significantly decreased. The results pertaining to the correlation analysis of these genes and metabolites are shown in Figure 10D.

The ferroptosis pathway occupied first place among the co-enriched pathways, with a *p*-value of less than 0.05, suggesting that this pathway may be significantly altered by RY-A. In particular, GSH levels were significantly elevated in RY-A-containing serum, and GSH expression levels are closely related to oxidative stress. These data suggest that RY-A may act by regulating oxidative stress and the ferroptosis pathway.

### 3.8 Results of analyzing the effect of RY-A on oxidative stress related indices

**FIGURE 11:**
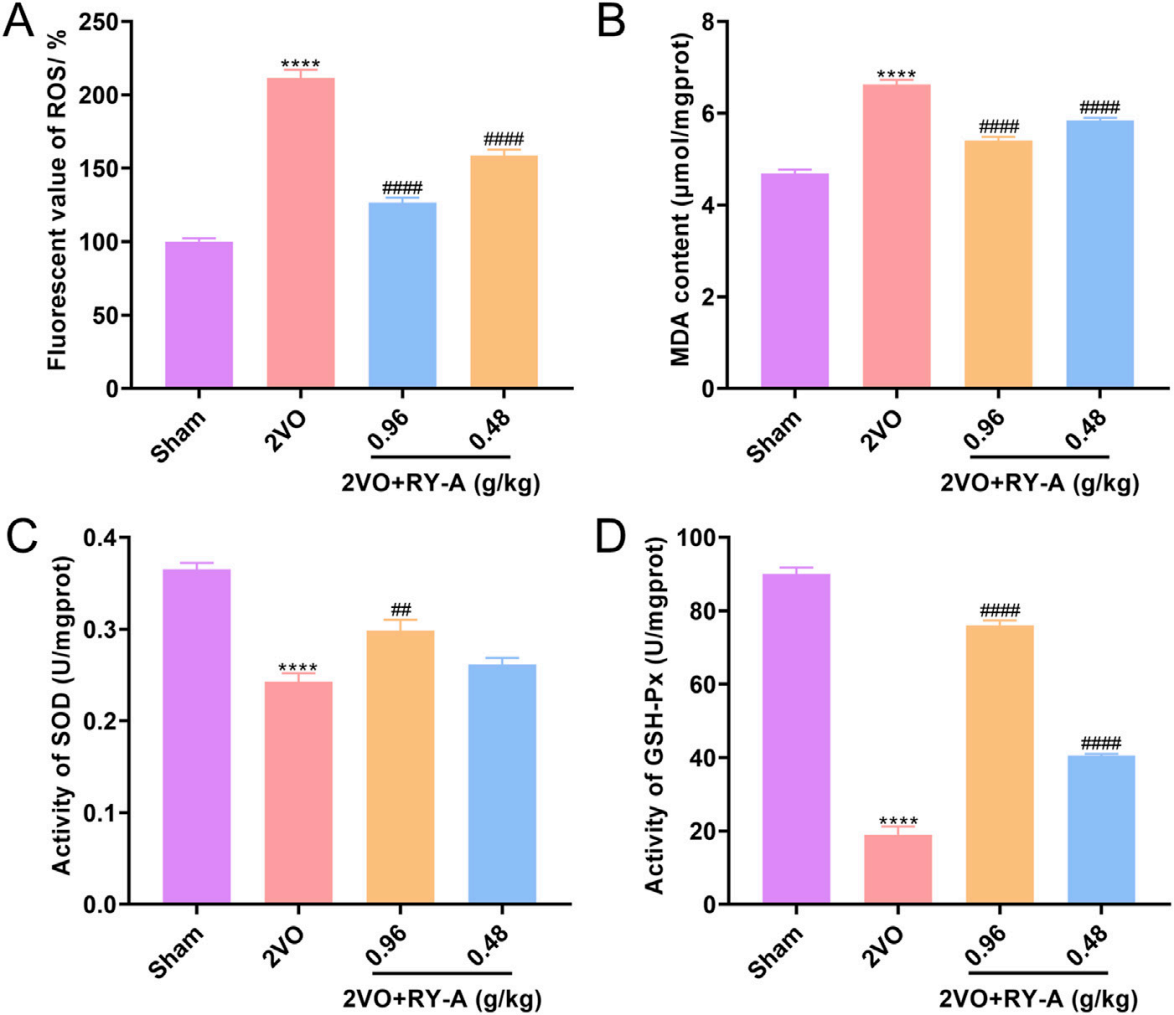
Inhibition of oxidative stress by RY-A. **(A)** ROS fluorescence values for each group of rats. **(B)** MDA content of rats in each group. **(C)** SOD activity in each group of rats. **(D)** GSH-Px activity in each group of rats. Data are expressed as mean ± SEM (n = 6). Compared to the Sham group, ****p < 0.0001; Compared to the 2VO group, ^*##*^ *p* < 0.01, ^*####*^ *p* < 0.0001. 2VO, 2-vessel occlusion; RY-A, *Rubia yunnanensis* alcohol extract.

A variety of kits were used to measure oxidative-stress-related markers, such as ROS, MDA, SOD, and GSH-Px, to see how well RY-A changes oxidative stress levels. We found that RY-A effectively stopped the production of pro-oxidant substances, such as ROS and MDA (Figures 11A, B), while greatly increasing the activity of antioxidant enzymes, such as SOD and GSH-Px (Figures 11C, D). Based on these indicators, the RY-A high-dose group demonstrated the best inhibitory effect. These results suggest that RY-A possesses notable antioxidant effects and is able to alleviate oxidative stress by enhancing the antioxidant defense system.

### 3.9 Western blot analysis for validation

The combined transcriptomic and metabolomic analysis showed that the ferroptosis pathway was significantly upregulated by RY-A treatment, and there is a close link between ferroptosis and CCH-induced VD (Fu et al., 2023). Thus, the presence of key proteins of the System Xc-/GSH/GPX4 pathway, including SLC7A11, SLC3A2, GSS, and GPX4, was validated by Western blot. These proteins play important roles in regulating oxidative stress and iron metabolism in cells.

**FIGURE 12:**
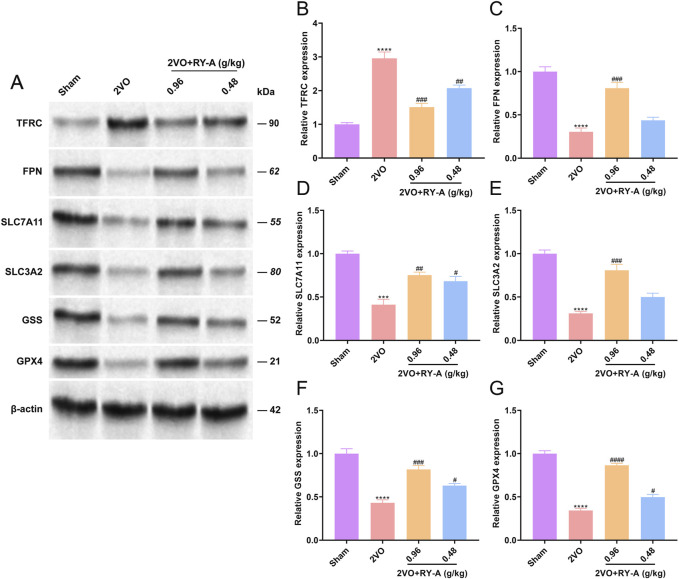
Regulation of intracellular iron ion homeostasis and System Xc-/GSH/GHX4 pathway by RY-A. Expression of **(A, B)** TFRC, **(A, C)** FPN, **(A, D)** SLC7A11, **(A, E)** SLC3A2, **(A, F)** GSS, **(A, G)** GPX4 in rat hippocampal tissues was detected by Western blot. Data are expressed as mean ± SEM (n = 3). Compared to the Sham group, ****p < 0.0001; Compared to the 2VO group, ^*##*^ *p* < 0.01, ^*####*^ *p* < 0.0001. 2VO, 2-vessel occlusion; RY-A, *Rubia yunnanensis* alcohol extract.

One of the features of ferroptosis is an imbalance in the intracellular labile iron pool (LIP), which leads to an increase in unstable iron ions. The main regulatory proteins of LIP include transferrin receptor (TFRC) and ferritin (FPN). To verify whether RY-A can regulate ferroptosis and related pathways, the expression levels of TFRC, FPN, and System Xc-/GSH/GHX4 pathway proteins in the rat groups were determined. The experimental results showed that the 2VO procedure significantly increased TFRC (Figures 12A, B) and suppressed FPN expression (Figures 12A, C), SLC7A11 (Figures 12A, D), SLC3A2 (Figures 12A, E), GSS (Figures 12A, F), and GPX4 levels (Figures 12A, G), in contrast to the Sham group. After RY-A treatment, the expression of TFRC was suppressed, whereas the expression of FPN, SLC7A11, SLC3A2, GSS, and GPX4 was activated, suggesting that RY-A promoted LIP homeostasis and activated the System Xc-/GSH/GPX4 pathway. These findings confirmed the role of RY-A in the regulation of the ferroptosis pathway at the molecular level and provided strong evidence for RY-A’s capacity as a potential neuroprotective agent.

### 3.10 Molecular docking analysis for validation

**FIGURE 13:**
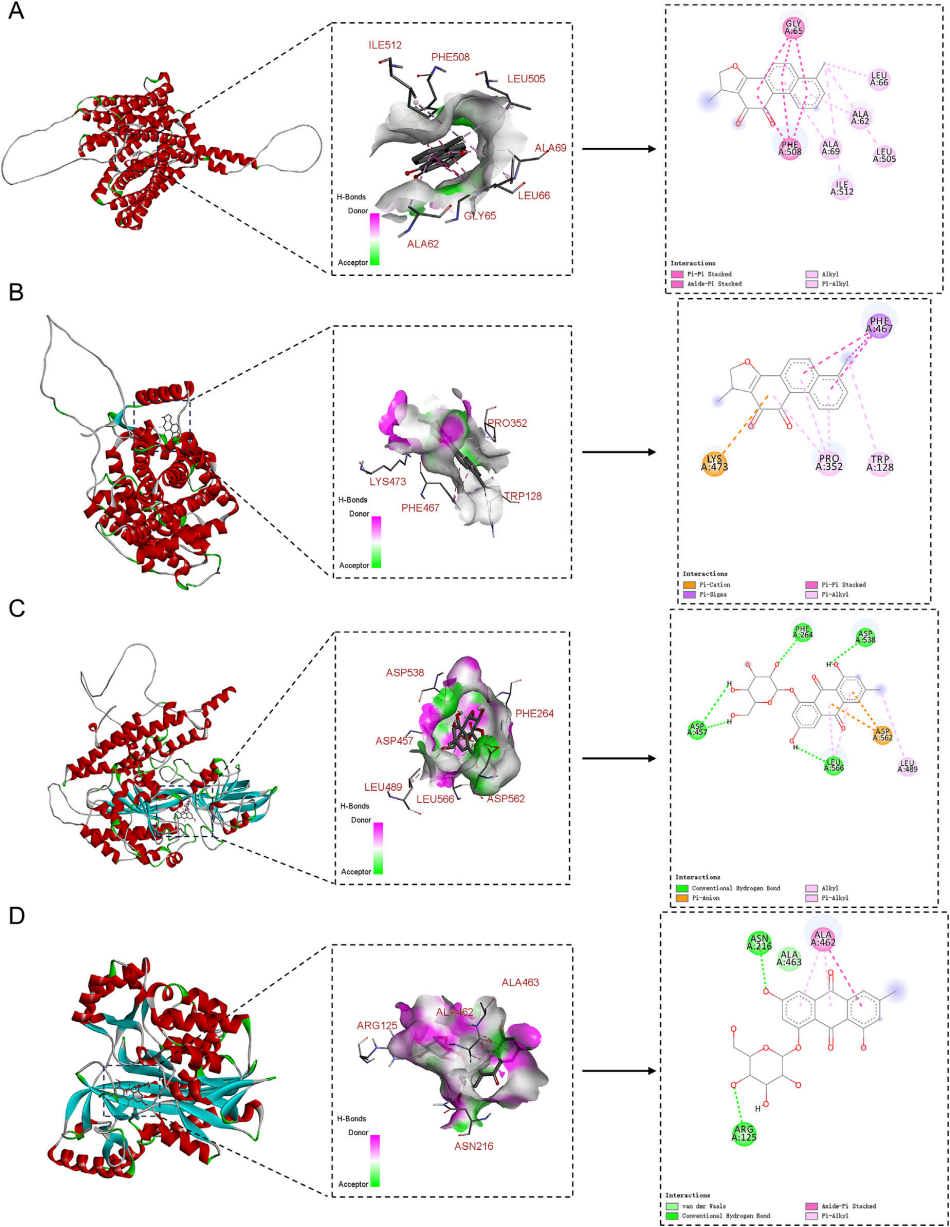
2D and 3D binding diagrams of molecular docking. **(A)** Dihydrotanshinone binding to FPN; **(B)** Dihydrotanshinone binding to SLC7A11; **(C)** Emodin 8-glucoside binding to TFRC; **(D)** Emodin 8-glucoside binding to GSS.

**FIGURE 14:**
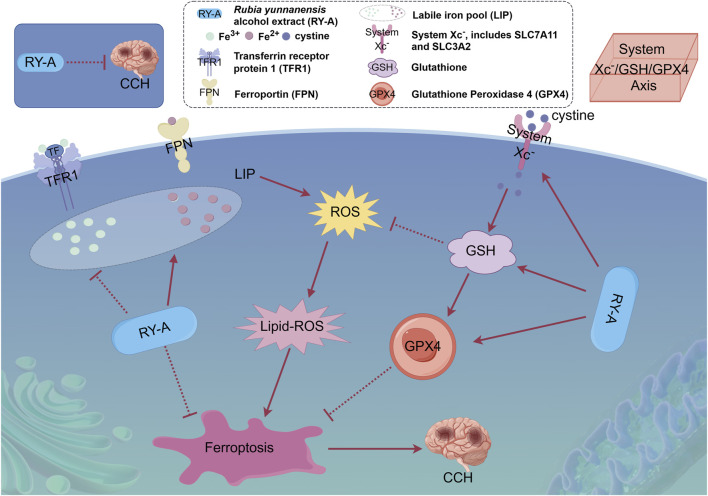
Overview of the mechanism of neuroprotective effects of RY-A in CCH rats. The protective effect of RY-A may be related to oxidative stress, labile iron pool, and regulation of the System Xc-/GSH/GPX4 pathway [By: Figdraw (www.figdraw.com)].

Lastly, this study explored the interactions between the potential active compounds in RY-A and ferroptosis-associated core target proteins using a molecular docking technique, and the relevant binding energy data are detailed in Supplementary Table S7. Dihydrotanshinone was found to have the strongest binding energies with FPN and SLC7A11 at −9.6 and −9.5 kcal/mol, respectively, suggesting that it may have high binding affinity. The binding energy of emodin 8-glucoside with TFRC and GSS followed closely at −9.5 kcal/mol. These results were visualized by two-dimensional and three-dimensional binding diagrams (Figure 13). In terms of intermolecular interactions, dihydrotanshinone partook in alkyl and Pi-alkyl interactions with ALA-69, ALA-62, LEU-66, ILE-512, and LEU-505 of FPN; and Pi-Pi stacked and amide-Pi stacked interactions with GLY-65 and PHE-508. Dihydrotanshinone partook in Pi-alkyl interactions with PRO-352 and TRP-128 of SLC7A11, Pi-Sigma and Pi-Pi stacked interactions with PHE-467, and a Pi-cation interaction with LYS-473. Whereas emodin 8-glucoside was involved in conventional hydrogen bond interactions with ASP-457, PHE-264, ASP-538, and LEU-566 of TFRC, Pi-anion interactions with ASP-562, and alkyl and Pi-alkyl interactions with LEU-489. Emodin 8-glucoside participated in conventional hydrogen bond interactions with ARG-125 and ASN-216 of GSS, van der Waals interactions with ALA-463, and amide-Pi stacked and Pi-alkyl interactions with ALA-462. These results suggest that these compounds inhibit oxidative stress and ferroptosis by interacting with specific amino acid residues of key target proteins, thereby modulating the System Xc-/GSH/GPX4 pathway and consequently attenuating the neurological damage and cognitive impairment in the CCH model (Figure 14).

## 4 Discussion

*Rubia yunnanensis* has a long history of use as an intervention in cardiovascular and cerebrovascular diseases, but its effects and mechanisms of action on CCH have not yet been reported. In the present study, it was demonstrated for the first time that RY-A has a therapeutic effect on CCH, as it ameliorated cortical and hippocampal neuronal damage and increased CBF, as well as improved spatial learning and memory ability in CCH rats. In the brain tissue of CCH rats, neuronal cells in the CA1, CA3, DG, and cortical regions were damaged to different degrees, and the morphology of these neuronal cells was improved after RY-A treatment. These observations were consistent with the results of the Morris water maze experiments, suggesting the ameliorative effect of RY-A on neuronal damage.

RY-A is a natural multi-component extract, thus the identification of its active compounds is challenging. Our previous work identified two compounds in RY-A, *Rubia yunnanensis* naphthoquinones A and *Rubia yunnanensis* quinone B, by HPLC analysis (Li et al., 2024). In this study, the UHPLC-HRMS technique was used to improve the specificity of the identification and characterize for the first time 511 blood-entry compounds of RY-A released into the blood (Supplementary Table S1). The reliability of these results was further verified by comparison with several standards, which included potentially active compounds such as rhein, emodin 8-glucoside, alisol A, dihydrotanshinone, and corosolic acid. Of these compounds, rhein has multiple anti-inflammatory, antioxidant, and anti-cancer effects. Reportedly, rhein is able to inhibit ferroptosis through the NRF2/SLC7A11/GPX4 signaling pathway, attenuate the damage caused by cerebral ischemia/reperfusion, and induce dose-dependent neuroprotective effects (Liu et al., 2023a). Emodin 8-glucoside has considerable antibacterial and anti-inflammatory activities. Wang et al. showed the protective effect of emodin 8-glucoside against ischemia-reperfusion-induced brain injury and glutamate-induced neuronal damage, as well as its high antioxidant properties (Wang et al., 2007). Alisol A was shown to lead to neurovascular protection in cerebral ischemic mice through the activation of the AKT/GSK3β signaling pathway; the compound attenuated the neurological deficits and cognitive deficits and inhibited IL-6 and IL-1β expression, in addition to promoting neuronal survival (Li et al., 2023). Wu et al. (2023) concluded that dihydrotanshinone is able to prevent ischemic stroke by activating Nrf2 and inhibiting ferroptosis. Their study revealed that dihydrotanshinone reduced ferroptosis through the activation of the Nrf2 signaling pathway, reduced the neurological damage in cerebral ischemic rats, and improved CBF and brain tissue microstructure. According to Zhang et al. (2024), corosolic acid can effectively protect the heart from cerebral ischemia-reperfusion injury in rat experiments. It functions by stifling oxidative stress and fostering mitochondrial autophagy, two processes intimately associated with ferroptosis. The effects of these compounds suggest that RY-A has the potential to treat cerebral ischemia.

In recent years, network pharmacology has provided a networked technical tool for research related to Traditional Chinese Medicine (Nam et al., 2024; Zhao et al., 2024). However, considering the limitations of incomplete databases and homogeneous substances, and the infeasibility of quantitative studies (Pohjala and Tammela, 2012; Luo et al., 2020), metabolomics and transcriptomics were selected in the present study to conduct a more reliable and quantitative analysis of the rat cortical tissues. The results more visually reflected the metabolites and genes that were significantly different before and after RY-A treatment. Between the two groups, a total of 53 DEGs and 107 DEMs were found in the current study. Transcriptomic analysis showed that, compared with 2VO group gene expression, most of the DEGs in the RY-A group were enriched in the calcium, TGF-beta, one carbon pool by folate, and ferroptosis signaling pathways. Calcium signaling is an important pathway that affects Ca^2+^ transport inside and outside neuronal cells and the normal physiological activities of neuronal cells. When excessive intracellular Ca^2+^ causes overloading, a series of Ca^2+^-dependent biochemical reactions, such as mitochondrial dysfunction, are induced, causing hypoxic neuronal injury and diminished memory and learning capacity (Tanovic and Alfaro, 2006). TGF-beta signaling is an important cytokine pathway associated with various pathological processes, incuding endothelial cell death, microglia activation, and neuronal loss. It also plays a role in tissue repair, which contributes to the improvement of vascular-related lesions and the enhancement of learning and memory abilities in patients with VD (Kandasamy et al., 2020). One carbon pool by folate pathway is associated with the biosynthesis of folic acid, a compound commonly used clinically as an adjunctive therapy for patients with dementia. This pathway exhibits neurotrophic effects and has been associated with CBF (Tu et al., 2023). Ferroptosis, a recently identified ferroptosis-dependent pathological process characterized by peroxidation and the inactivation of GSH and GPX4, has been shown to be induced by the CCH-mediated inactivation of System Xc-associated transporter proteins (Fu et al., 2023). Clearly, these pathways play key roles in neuronal function and tissue repair and are closely related to the pathophysiological process of CCH.

Our metabolomic analysis showed that, when compared to 2VO group expression, most of the DEMs in RY-A were enriched in fatty acid biosynthesis (ko00061), biosynthesis of unsaturated fatty acids (ko01040), ferroptosis (ko04216), and glutathione metabolism (ko00480). The role of fatty acids is like a double-edged sword, with the biosynthesis of fatty acids, especially unsaturated fatty acids, being one of the key causes of ferroptosis production (She et al., 2020). Thus, omega-3 polyunsaturated fatty acids for dietary use have been suggested as a non-medical alternative for improving brain function and dementia (Burckhardt et al., 2016). Furthermore, a study by Mukamal (2020) found that circulating levels of nonesterified fatty acids can contribute to the development of cardiovascular diseases and can be used as a preventive indicator f of cardiovascular disease in healthy populations. Glutathione metabolism maintains the normal immune system of the body; GSH is considered the most abundant antioxidant, and a decrease in its level marks the generation of oxidative stress (Detcheverry et al., 2023). In turn, activation of GPX4 prevents lipid peroxidation and ferroptosis while inhibiting tumor growth (Xu et al., 2021). These metabolites play important roles in brain cell function and oxidative stress, with the regulation of glutathione metabolism being critical for maintaining redox homeostasis.

In the integrated transcriptomics and metabolomics investigations, the ferroptosis pathway ranked first among the co-enriched pathways, and the level of GSH was significantly increased after RY-A treatment. GSH is strongly linked to the progression of several neurodegenerative disorders, including Alzheimer’s and Parkinson’s diseases, and is regarded as a potential therapeutic target (Aoyama, 2021; Shi et al., 2022). Our prior research showed the inhibitory impact of RY-A on the oxidative stress generated by oxygen-glucose deprivation/reoxygenation (OGD/R) in HT22 neural cells (Cheng et al., 2024). Our examination of the combined omics data suggested that RY-A may function by mitigating oxidative stress and ferroptosis.

The results of the oxidative stress-related kit assay showed that the antioxidant enzymes SOD and GSH-Px were inhibited and ROS and MDA levels were elevated in CCH rats compared to Sham rats. These indices were reversed by RY-A treatment of CCH rats compared to untreated CCH rats, with the results converging toward those of the Sham group and optimal effects seen in the high-dose RY-A group. The results were clear in suggesting that RY-A treatment inhibited the occurrence of oxidative stress.

An imbalance in intracellular LIP is one of the most important reasons for the emergence of ferroptosis, and two key ferric ion transport proteins that regulate the balance of the iron pool are TFRC and FPN (Rochette et al., 2015; Zheng and Conrad, 2020). The System Xc-/GSH/GHX4 pathway, an important pathway linking GSH to ferroptosis, has been shown to ameliorate the neuronal loss induced by cerebral ischemia-reperfusion injury in previous reports (Xu et al., 2023). The main proteins of System Xc- are SLC7A11 and SLC3A2 (Wu et al., 2024). Therefore, the effects of RY-A on the iron-metabolizing proteins within the System Xc-/GSH/GHX4 pathway were further explored. The results of Western blot experiments showed that the expression of TFRC was suppressed after RY-A treatment. The relevant literature demonstrates that TFRC is a signature protein for the uptake of ferric ions into cells, and inhibition of its expression prevents ferroptosis production (Xu et al., 2023). Furthermore, we found that RY-A activated the expression of FPN, which is considered to be a major intracellular iron storage protein. In addition, ferroptosis production involves the autophagic degradation of FPN, hence blocking its degradation could be used to resist ferroptosis (Chen et al., 2020). Our research additionally showed that giving rats RY-A increased the levels of SLC7A11, SLC3A2, GSS, and GPX4 proteins in the System Xc-/GSH/GHX4 pathway. SLC7A11 and SLC3A2 are the principal proteins of System Xc-, a cystine/glutamate reverse transporter essential for the cellular uptake of cystine. Cystine is subsequently reduced to cysteine, facilitating the synthesis of GSH (Ishii et al., 1992). Liu et al. (2019) showed that increasing the expression of SLC7A11 by inducing the expression of the deubiquitinating enzyme OTUB1 protected cells from ferroptosis. The expression of GSS was also activated by RY-A treatment in this study; GSS is a major synthase of GSH, and increasing the expression of GSH was observed to inhibit ferroptosis (Rochette et al., 2022). The dysfunction of GPX4, a glutathione peroxidase that degrades intracellular lipid peroxides into non-toxic alcohols, is considered a key feature of ferroptosis (Cao and Dixon, 2016; Chen et al., 2021). Research found that the occurrence of ferroptosis in mice was closely linked to the deletion of SLC7A11 and GPX4 expression, which further led to cognitive impairment and early embryonic death (Koppula et al., 2021).

Clearly, the above evidence supports our conjecture that RY-A inhibits oxidative stress, promotes intracellular LIP homeostasis and activates the System Xc-/GSH/GHX4 pathway.

However, there are several limitations to this study. Initially, the study primarily relied on animal models and was lacking clinical validation, and thus its translational application requires further confirmation. Additionally, although multiple active compounds in RY-A were identified, their relative contribution to the overall therapeutic effect remains unclear. Future research should aim to further clarify the roles and interactions of these compounds.

## 5 Conclusion

The potential therapeutic impact of RY-A on CCH and its mechanism was examined using an integrated approach combining serum pharmacochemistry, metabolomics, and transcriptomics, with verification through animal experiments. The findings of this provide a scientific foundation for the pharmacological effects of *Rubia yunnanensis* and offer novel perspectives on advanced preventive and treatment techniques for cerebral ischemia.

## Data Availability

The original contribution presented in this study is included in the article/Supplementary Material, and the raw RNA-seq data are freely available in the NCBI database under accession no. PRJNA1174397.

## Abbreviations

BPC, base peak chromatogram; CCH, chronic cerebral hypoperfusion; CBF, cerebral blood flow; DEGs, differentially expressed genes; DEMs, differentially expressed metabolites; FPKM, fragments per kilobase of transcript per million fragments mapped; GO, Gene Ontology; HE, hematoxylin and eosin; KEGG, Kyoto Encyclopedia of Genes and Genomes; LIP, labile iron pool; MRM, multiple reaction monitoring; OPLS-DA, orthogonal partial least squares discriminant analysis; RY-A, Ethanol extraction from *Rubia yunnanensis*; UHPLC-HRMS, ultra-high-performance liquid chromatography high-resolution mass spectrometry; VD, vascular dementia; XIC, extracted ion chromatograms; 2VO, two-vessel occlusion.
